# Bioresponsive Nanomedicine: The Next Step of Deadliest Cancers' Theranostics

**DOI:** 10.3389/fchem.2020.00257

**Published:** 2020-04-09

**Authors:** Yuqiang Mao, Xiaoying Liu

**Affiliations:** ^1^Department of Thoracic Surgery, Shengjing Hospital, China Medical University, Shenyang, China; ^2^Department of Breast Surgery, The First Affiliated Hospital of China Medical University, Shenyang, China

**Keywords:** bioresponsive nanomedicine, nanoparticles, theranostics, breast cancer, lung cancer, prostate cancer

## Abstract

Among all cancers, lung, breast, and prostate carcinoma are the three most fatal cancers. Although general therapeutic strategies and existent nanomedicine have been applied in relating cancer treatments, the side effects and potential damage induced by the off-target effect greatly lower the therapeutic efficiency. Recently, an increasing number of bioresponsive nanomaterials is recruited in fighting these deadliest cancers. Therefore, these latest bioresponsive nanomedicine are summarized in the current review. More specifically, the various novel nano-agents that could selectively respond to specific bio-conditions in malignant areas (e.g., pH, temperature, enzyme, Redox, elevated copper ion, etc.) are discussed in detail for their applications in cancer imaging (e.g., fluorescence, NIR, and MRI, etc.) and therapy (e.g., antiangiogenesis, chemotherapy, photothermal, and chemodynamic therapy, etc.). The development of next-generation of bioresponsive nanomedicine and challenges involved are further discussed for future design.

## Introduction

Cancer is a disease caused by gene mutation, leading to an uncontrolled cell division. These abnormal cells could easily generate malignant lesions and even metastasize to other organs, greatly threatening the patient's health. As the second leading cause of deaths, cancer contributed to around 10 million deaths in 2018 globally, with about 18.1 million new cases (Bray et al., 2018). Among all the cancer types, lung, breast, and prostate cancer are the deadliest carcinomas for people, contributing to 18.4, 11.6, and 7.1% of all the cancer-related deaths, respectively (Bray et al., 2018). With the support of various diagnosis technologies including positron emission tomography (PET), magnetic resonance imaging (MRI), and computed tomography (CT), surgery, radiotherapy, chemotherapy, and hormone therapy are generally applied to treat patients with cancer, including those three deadliest carcinomas. However, most strategies fail to detect and eliminate the cancerous cells efficiently, which could lead to tumor occurrence and threat to the patient's life. In addition, accompanying side effects can not be completely avoided, causing various adverse effects ranging from vomiting to asthenia (Oun et al., 2018). Although immunotherapy could be the best choice for preventing these situations, the high cost could be only afforded by a small portion of patients.

With the development of nanotechnology, functional agents could be uniformly synthesized at nano-size, showing great potential in the biomedical application as nanomedicine. These nanomaterials could perform as anti-cancer agents by carrying effective cargos (e.g., anti-cancer drugs or monoclonal antibody, etc.) and processing directly therapeutic effects (e.g., photothermal and photodynamic therapy, etc.). Compared with general treatments, nanomedicines greatly increase the efficiency of therapy (e.g., the increased loading compacity, prolonged circulation of drug and combined therapies, etc.) and limit side effects (e.g., encapsulated anti-cancer drugs and specific tumor targeting, etc.). To date, various nanomedicines have been approved by the FDA for cancer therapy, such as paclitaxel albumin-bound nanoparticles (Abraxane) and liposomal irinotecan (Onivyde) (Ventola, 2017). Although these nano-agents exhibit desirable anti-cancer function, the constantly active cytotoxicity would potentially cause indiscriminate damage to normal tissues due to the off-target effect. Recently, a growing number of novel nanomaterials that are specifically responded to biological factors (e.g., acidity, enzyme, redox, temperature, and copper ion, etc.) within tumor area are recruited for fighting these deadliest cancers via imaging (e.g., fluorescence, near infrared (NIR), and MRI, etc.) and therapy (e.g., antiangiogenesis, chemotherapy, photothermal, and chemodynamic therapy, etc.). Therefore, these recent bioresponsive nano-platforms that have been investigated in lung, breast, and prostate cancer theranostics are highlighted in the current review. Meanwhile, the future direction and challenges involved are discussed as well, aiming to offer an overview of the development of smart nanomedicine in treating these deadliest carcinomas.

## Current Situation of Lung, Breast and Prostate Cancer

As the essential organ for body function, the lung is strongly associated with other systems, which make it very vulnerable to illness, such as cancer. The lung carcinoma is a life-threating disease, especially those metastasized from another part of the body, which generally indicated the late stage of cancer, such as advanced breast and prostate cancer. Normally, the 5-year life expectancy of patients with distant lung cancer is only about 5% (Torre et al., 2016). As another major carcinoma, breast cancer is the most common cancer among females. In 2020, there are an estimated 325,000 females who will be diagnosed with invasive or non-invasive breast cancer. Comparatively, prostate cancer contributes to around 366,000 men deaths and 1.6 million new cases annually (Pernar et al., 2018). Although the people with increased risks (e.g., aging and family histology) will be easier to develop these fatal cancers, there is an increasing trend of young patients diagnosed with lung and prostate cancer overall the world (Salinas et al., 2014; Liu et al., 2019a).

## General Therapeutic Strategies

To deal with these deadliest cancers, general treatments such as surgery, chemotherapy, and radiotherapy have been recruited as routine strategies for years. Although current clinical diagnosis could promote outcomes of these treatments in the early stage, the efficiency of most therapies is limited for late-stage or advanced cancer. Recent years, the precision medicine based on individual genetic information provide effective therapy for patient via specific targeting such as the blockage of certain growth factor receptor (Dienstmann et al., 2017 #189). Although these precision therapies could work well in most cancers with general targets, its feasibility in mutated cancer types such as non-small-cell lung cancer of adenocarcinoma with EGFR mutation (~29.3% of all) or triple-negative breast cancer (11.2% of all) is strongly restricted by the availability of small molecular or monoclonal antibody (Midha et al., 2015 #190, Tan and Dent, 2018 #191). Due to these, there is an urgent need of a novel therapeutic approach for treating developed and mutated cancers.

## Current Status of Nanomedicine for Lung, Breast, and Prostate Cancer Therapy

Nanomedicine, as an advanced technique has been gradually applied in fighting cancer, especially lung, breast, and prostate cancers. Since 2018, more than 20 nano-sized medicine have been parenteral in the market, while seven of them are designed for cancer therapy (Flühmann et al., 2019). Currently, there are about 27 clinical trials associating with the nanoparticles based breast cancer therapy are active. These studies focus on imaging and treating various breast carcinoma ranging from triple-negative breast cancer (TNBC) to metastatic breast cancer. A series of nanomedicines including FDA-approved paclitaxel albumin-bound nanoparticles (Abraxane), lipid nanoparticles (mRNA-2752), curcumin/doxorubicin encapsulating nanoparticles (Imx-110), quantum dots and silica nanoparticle (cRGDY-PEG-Cy5.5-C dots) have been recruited in clinic studies, showing the great potential of nanomedicine in dealing breast cancer. In comparison, there are about 13 and 9 active clinical trials related to lung and prostate cancer, respectively. Notably, either the mostly-applied Abraxane or newly-designed cyclodextrin-based polymer (CRLX101) is constantly functional that will potentially damage the normal cells. Therefore, more efficient and safe cancer therapy could be provided by the novel nano-platforms with therapeutic functions that could smartly be activated by specific conditions, such as bioresponsive nanomedicine.

## Bioresponsive Nanomedicine

Certain biological factors including acidic extracellular environment, specific enzymes, elevated redox and cooper ion, etc. are well-identified in the tumor micro-environment (TME). Base on these factors, a series of advanced nanoplatforms with TME-activated functions have been successfully developed for treating these three fatal cancers (Table 1). In a comparison of current nanomedicines that generally exhibit constantly-activating functions (e.g., cytotoxicity of anti-cancer drugs), the therapeutic effects of bioresponsive nano-agents could be smartly triggered in TME, which efficiently avoids most of the adverse influence caused by miss-targeting.

**Table 1:** Recent advanced bioresponsive nanomedicine used in treating lung, breast, and prostate cancer.

**Bioresponsive factor**	**Type of nanomaterial**	**Nanomedicine**	**Size (nm)**	**Application**	**Cancer**	**References**
Acidity	Polymer	PWMs	19.9 ± 1.9 × 50–200	siRNA delivery	Breast cancer with lung metastasis	(He et al., 2016 #152)
Acidity	Polymer	NP15	<100	siRNA delivery	Breast cancer	(Saw et al., 2019 #157)
Acidity	Silica	TPZ@HHSN-C/P-mAb	142.5 ± 1.3	USI and MRI—SDT and BRT	Prostate cancer	(Wang et al., 2019 #159)
Redox and acidity	Polymer	P-RUB micelles	49 ± 0.26	Chemo	Taxane resistant prostate cancer	(Lin et al., 2019 #163)
Redox and acidity	Silica	ECMI	220.0 ± 3.5	PDT and Chemo	Erlotinib-resistant EGFR-mutated NSCLC	(Zhang et al., 2019 #164)
Redox	Nanozyme	Lipo-OGzyme-AIE	122.5	PDT	Breast cancer with lung metastasis	(Gao et al., 2020 #168)
Enzyme and Redox	Gold NCs	mCAuNCs@HA	150	PDT and Chemo and Immuno	Breast cancer with lung metastasis	(Yu et al., 2019 #174)
Enzyme	Polymer	HACE	132	NIRF and PAI—PDT	Lung cancer	(Li et al., 2016 #173)
Enzyme	Polymer	WINNER	16	mAbs delivery	Lung cancer	(Li et al., 2019 #178)
Enzyme	Polymer	Self-assembled polymer	93	Chemo	Lung cancer	(Yang et al., 2016 #179)
Copper and acidity	Polymer	RPTDH	200	Antiangiogenic and Immuno	Metastatic breast cancer	(Zhou et al., 2019 #181)
Copper	Silica	Imi-OSi	<6	Antiangiogenic and TVO	Breast and lung cancer	(Yang et al., 2019a #182)
Thermal and acidity	Polymer	mPEG-PAAV	174.5	NIRF and PAI—PTT and Chemo	Breast cancer with lung metastasis	(Yang et al., 2018 #184)

*USI, ultrasound imaging; MRI, magnetic resonance imaging; SDT, sonodynamic therapy; BRT, bioreductive therapy; Chemo, chemotherapy; PDT, photodynamic therapy; Immuno, immunotherapy; NIRF, near infrared fluorescence; PAI, photoacoustics imaging; TVO, tumor vascular obstructing*.

## pH-Responsive Nanomedicine

**Figure 1:**
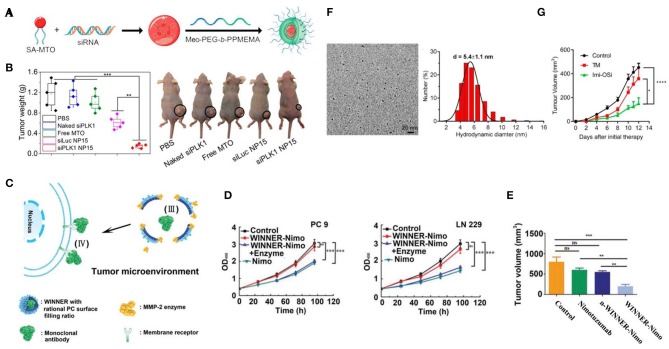
Bioresponsive nanomedicine for lung, breast, and prostate cancer therapy. **(A)** Scheme of synthesis of SA-MTO (NP15); **(B)** MDA-MB-231 tumor weights from nude mice xenograft model that were treated with different groups and representative photograph tumor-bearing mice at day 18 [(Saw et al., 2019) #157] (Copyright 2019, reproduced with permission from American Chemical Society). **(C)** Schematic illustration of WINNER coating with PC ratio for extracellular delivery of mAb; **(D)** The antitumor efficacy of WINNER-Nimo in LN 229 and PC 9 cells; **(E)** Tumor volume from different treatment groups [(Li et al., 2019) #178] (Copyright 2019, reproduced with permission from Wiley). **(F)** TEM image and DLS of Imi-OSi; **(G)** 4T1 tumor inhibition efficiency of TM (copper chelator) and Imi-OSi (Yang et al., 2019b #182) (Copyright 2019, reproduced with permission from American Chemical Society).

With excessive aerobic glycolysis, the extracellular area around cancerous cells is packed with lactic acid, showing an acidic environment with pH ranging from 6.5 to 6.9 (Kato et al., 2013). As the major tumor feature, various nanomaterials including polymer (Kato et al., 2013; He et al., 2016; Xu et al., 2017; Zhao et al., 2017; Shen et al., 2018; Saw et al., 2019), silica (Wang et al., 2018, 2019) and upconversion (Qiao et al., 2017) nanoparticles were designed for smart drug delivery via pH-response. With excellent pH-responsive features (e.g., via structural or solubility change), polymer-based nano-platforms demonstrate a great advantage in pH-triggered drug release (Kocak et al., 2017). By coating with pH-sensitive mPEG-b–PDPA_20_, succinobucol (SCB), vascular cell adhesion molecule-1 (VCAM-1) inhibitor could efficiently escape from micelles (PWMs) at TME, and inhibit the lung metastasis of breast cancer tumors for around ~6.25 and 4.5 times, respectively, in comparison with saline and SCB groups (He et al., 2016). Besides, by combing enzyme-induced feature (esterase), Saw et al. successfully synthesized an N15 polymer nanoparticle (<100 nm) consisting of a core (siRNA and amphiphilic cationic mitoxantrone, MTO) and pH-responsive PEG shell (Saw et al., 2019) (Figure 1A). The siRNA of Polo-like kinase 1 (PLK1) (more than 90%) would be only released after a two-step decomposition caused by acidic pH and esterase in the tumor area, which efficiently inhibited ~70% of PLK1 expression and around 2-fold of MDA-MB-231 tumor growth within 18 days (Figure 1B). Meanwhile, a silica based multi-module theranostic platform (HHSN-C/P-mAb) was developed by Wang et al. for imaging (US and MRI) and treating (sonodynamic and bioreductive therapy) prostate cancer (Wang et al., 2019). This acidic-degraded silica nanomedicine was able to target PC3 tumors (via modified monoclonal antibody of prostate stem cell antigen) and smartly release tirapazamine (TPZ) at TME, eventually inhibiting more than 91.5% tumor growth with US irradiation.

## Redox-Responsive Nanomedicine

As other major factors, the concentrations of reactive oxygen species (ROS) and glutathione (GSH) are extremely higher in TME (Cook et al., 2004), which allow different nano-agents to be applied for treating aggressive cancers including taxane resistant prostate cancer, erlotinib-resistant EGFR-mutated NSCLC cells and TNBC via redox-induced therapeutic functions (He et al., 2018; Dai et al., 2019; Hu et al., 2019; Lin et al., 2019; Liu et al., 2019b; Yang et al., 2019a; Zhang et al., 2019; Gao et al., 2020). In the combination of acidic and GSH sensitive features, a novel P-RUB micelle was designed for co-delivering docetaxel (DTX) and rubone (RUB) in fighting taxane resistant prostate cancer (PC3-TXR) (Lin et al., 2019). As-prepared nanomedicine (49 ± 0.26 nm) could quickly release both cargos (~100%) at acidic conditions (pH = 5) when GSH (10 mM) was presented. With two-step triggered therapy, the proliferation of PC3-TXR tumors was efficiently suppressed for ~50% compared to other groups. Similarly, pH-responsive zinc oxide quantum dots (ZnO QDs) and disulfide bond linked chitosan (Cs) were employed as triggers in ECMI silica nanoparticles for lung cancer therapy (Zhang et al., 2019). ECMI carrying ICG and erlotinib exhibited specific distribution and effective inhibition for EGFR mutated NSCLC tumors with the NIR irradiation. Most recently, Gao et al. developed a hypoxia-tropic nanozymes as theranostic nanomedicine for suppressing orthotopic breast cancer growth and lung metastasis (Gao et al., 2020). Significantly, the MnO_2_ core inside ferritin nanocages (FTn) catalase the H^+^ and H_2_O_2_ into O_2_ that could be generated into toxic ROS by AIE modules under the irradiation. These reactions could effectively alter the TME (e.g., neutralization of hypoxic environment, with ~51.9 ± 8.5% reduction of hypoxic tissue) and prohibit the proliferation and metastasis of breast cancer with only ~5% metastasis compared to PBS group.

## Enzyme-Responsive Nanomedicine

Besides, various enzymes are strongly associated with cancerous cells. It has been found that the excess hyaluronidase could be found within malignant tissues including lung, prostate, and breast carcinoma (Mcatee et al., 2014). More importantly, the presentation of hyaluronidase could accelerate the development of cancer progression. Li et al. were inspired by this tumor-related enzyme and developed a hyaluronidase-activated theranostic micelles (HACE NPs) with hyaluronic acid (HA) and chlorin e6 (Ce6) (Li et al., 2016). This polymer (132 nm) could produce both A549 tumor imaging (including NIR fluorescence and photoacoustic imaging) and photodynamic therapy (PDT) via the Ce6 that was released by hyaluronidase. Similarly, a HA- and ROS-responsive nano-agent (mCAuNCs@HA) was successfully recruited in treating breast cancer and lung metastasis via PDT and the blockage of checkpoint (Yu et al., 2019).

Matrix metalloproteinase (MMP) is another type of tumor-specific enzyme. Notably, a significant difference could be observed between malignant and normal lung tissues in terms of the expression of MMPs (Merchant et al., 2017), showing great potential of MMPs as the bioresponsive factor for lung cancer theranostics. Recently, a series of MMPs-responsive (especially MMP2/9) nanomedicines were developed in therapy against lung cancer (Van Rijt et al., 2015; Yang et al., 2016; Battistella et al., 2019; Li et al., 2019). For instance, Yang et al. prepared self-assembled nano drugs by conjugating an MMP-2-cleavable peptide, which allowed this nanomedicine selectively to deliver camptothecin (CP) and trans-retinoic acid (RA) antitumor drugs to cancerous cells with high expression of MMP-2 (Yang et al., 2016). In the comparison of typical intracellular drug delivery, Li et al. designed a novel nano-vehicle (WINNER) for specific extracellular delivery (Figure 1C). With MMP-2-responsive peptides and controlled surface filling ratio (50.5–58.3%) of phosphorylcholine (PC), WINNER could efficiently protect and release loading nimotuzumab to the lung tumor (PC-9 and LN-229) extracellular area, showing highest antitumor effect in compared with free nimotuzumab (Figures 1D,E).

## Other Bio-Responsive Nanomedicine

An increasing number of studies have found that elevated serum copper ion was associated with various cancers and strongly related to the stage and progression of carcinoma, such as breast cancer (Denoyer et al., 2015). As a promising tumor stimulus, copper ion also plays a key role in tumor angiogenesis. In most recent, two copper-chelator based nanomedicines have been synthesized for lung and breast cancer therapy (Yang et al., 2019b; Zhou et al., 2019) (Figure 1F). In addition to the anti-angiogenesis induced by chelation of Cu^2+^, these smart nanoparticles caused further anti-tumor effects via tumor vessel obstruction (e.g., aggregation of nanochelators) and TLR-mediated immune cells stimulation (with TLR7 and TLR8 agonist), respectively (Figure 1G).

Meanwhile, several temperature-responsive nanomedicines were also developed for prostate and breast carcinoma therapy (Wadajkar et al., 2013; Yang et al., 2018). These nano-composites are sensitive to the change of temperature (40–43°) and will release the cargos at lower or upper critical solution temperature, eventually triggering the anti-cancer effects via chemotherapy or combined therapy.

## Conclusion and Future Outlook

The deadliest cancers, lung, breast, and prostate cancers cause thousands of deaths annually, while the efficiency of general strategies is limited, especially for those with drug resistance or genetic mutations. In comparison, the bioresponsive nanomedicine has shown great potential in treating deadliest cancers via the smartly-triggered (e.g., pH, Redox, Enzyme, Copper ion, temperature) functions. Since now, a series of these nano-agents have been successfully developed and shown promising outcomes. Undeniably, the bioresponsive nanomedicine would be the next step of theranostics for these deadliest cancers. For future development, we believe there are several issues could be considered. (1) In consideration of the different TME, specific designs of bioresponsive nanomedicine are highly recommended. For instance, with significantly elevated MMPs, the MMP-activated nanomedicine is more promising and feasible in lung cancer compared with others. (2) The novel direction such as extracellular delivery could be further designed for treating TME. (3) Plural triggers (e.g., pH and Redox, pH and Enzyme) for function activation could be achieved by incorporating functional nanomaterials, such as polymers, which would provide more precision therapy. (4) Meanwhile, multiple functions (e.g., image-guided surgery and therapy) are demanded for the future bioresponsive nanomedicine. (5) Although bioresponsive nanomedicines are more smarted and precise compared with general therapy, specific training of handling this type of novel medicine (e.g., the pH- and Redox-responsive nanomedicine) during manufacture, delivery, storage, and therapeutic process may be required, which may potentially increase the cost for treatment. Therefore, the re-design of current nanomedicine as bioresponsive agents or collaboration between industry and research and could be options for reducing the expense in the quality control and further training.

## Author Contributions

YM wrote the manuscript with the help and guidance of XL.

### Conflict of Interest

The authors declare that the research was conducted in the absence of any commercial or financial relationships that could be construed as a potential conflict of interest.
